# The interaction between diet quality and cigarette smoking on the incidence of hypertension, stroke, cardiovascular diseases, and all-cause mortality

**DOI:** 10.1038/s41598-024-62616-9

**Published:** 2024-05-29

**Authors:** Mostafa Norouzzadeh, Farshad Teymoori, Hossein Farhadnejad, Nazanin Moslehi, Seyedeh Tayebeh Rahideh, Parvin Mirmiran, Fereidoun Azizi

**Affiliations:** 1 Nutrition and Endocrine Research Center, Research Institute for Endocrine Sciences, Shahid Beheshti University of Medical Sciences, Tehran, Iran; 2 Department of Nutrition, School of Public Health, Iran University of Medical Sciences, Tehran, Iran; 3 Endocrine Research Center, Research Institute for Endocrine Sciences, Shahid Beheshti University of Medical Sciences, Tehran, Iran

**Keywords:** Cardiovascular diseases, Nutrition disorders, Epidemiology, Risk factors

## Abstract

This study aimed to examine the interaction between diet quality indices (DQIs) and smoking on the incidence of hypertension (HTN), stroke, cardiovascular diseases, and all-cause mortality. We prospectively followed 5720 participants and collected dietary data via a validated food frequency questionnaire to calculate DQI-international (DQI-I) and DQI-revised (DQI-R). Considering an interaction analysis, we classified participants based on diet quality (median: higher/lower) and smoking status. Over 9 years of follow-up, higher diet quality scores were associated with a lower risk of stroke and mortality. While current smokers had a higher risk of stroke and mortality but had a lower risk of developing HTN. Compared to the current smokers with lower diet quality, nonsmokers with higher diet quality according to the DQI-I [HR 0.24; 95% CI (0.08, 0.66)], and DQI-R [HR 0.20; 95% CI (0.07, 0.57)] had a lower risk of stroke. Moreover, the lower risk of mortality was more evident in nonsmokers with higher DQI-I [HR 0.40; 95% CI (0.22–0.75)] and DQI-R scores [HR 0.34; 95% CI (0.18–0.63)] compared to nonsmokers with lower diet quality. While higher DQI-I and DQI-R scores were associated with a lower risk of stroke and mortality, this beneficial effect may be negated by smoking.

## Introduction

Non-communicable diseases (NCDs), particularly cardiovascular diseases (CVDs), represent the predominant global cause of mortality^1^. Iran has a high prevalence rate of CVD (> 9000 cases per 100,000 individuals), accounting for almost half of the mortalities and contributing to 20–23% of the overall disease burden^2^. Notably, hypertension (HTN) has emerged as the leading contributor to CVDs incidence, affecting one in every four Iranians^3,4^. While smoking and poor diet are recognized as the primary contributors to mortality risk^5^, it has been shown that CVDs can be managed through dietary and lifestyle behaviors^6,7^.

Smoking contributes to nearly 25% of annual stroke incidents and approximately 15% of stroke-related mortality^8,9^. Cigarette smoking has a pronounced effect on males, adults ranging from 18 to 44 years old, individuals with lower socioeconomic status, and residents of developing nations^10,11^. Evidence suggests that smokers with poorer nutritional status have a twofold higher risk of developing CVDs^12,13^. Smokers often adopt unhealthy dietary habits, like lower intake of fruits and vegetables and higher consumption of fats and alcohol^14,15^. The mutually influencing disease-causing mechanisms common to both smoking and poor diet quality can interact progressively, indicating the higher potential risk of NCDs over time^16^. Meanwhile, the optimal method for considering associations and interactions among dietary components is to assess overall diet quality^17^. Dietary quality indices (DQIs) offer a thorough approach to assessing dietary behaviors with an emphasis on dietary variety, dietary moderation, balanced nutrient intake, and diverse food choices^18^.

Several studies have individually examined the impact of dietary factors or smoking on health risks. Still, there is a notable gap in research exploring how these factors might interact and influence health outcomes. Previous studies have indicated that a higher score on the DQIs is linked to lower levels of inflammatory markers, which are known risk factors for CVDs^19^. Furthermore, a higher score on these indices has been associated with higher levels of high-density lipoprotein-cholesterol (HDL-C), which is a protective factor against CVDs^20^. However, few studies have explored the relationship between these indices and the risk of CVDs. Additionally, while smoking is a well-known risk factor for CVD incidence, some studies have suggested a lower risk of HTN among smokers^21^. Besides, studies examining the diets of smokers predominantly focused on individual dietary components rather than assessing overall dietary patterns^22,23^. To the best of our knowledge, there are no studies investigating the interaction between a high-quality diet as a protective factor and smoking as a risk factor for CVDs risk, particularly in the Middle East and North Africa (MENA) region.

Considering the conflicting results and limited available data, this study can contribute to shaping more effective recommendations and dietary guidelines for the management of CVDs outcomes. Hence, this study was conducted to investigate the associations between diet quality, smoking, and their interactions with the incidence of HTN, stroke, CVDs, and all-cause mortality.

## Results

The mean age ± SD of the participants recruited for the study of cardiovascular events was 46.42 years, and 44.8% were men. The baseline characteristics of the study participants based on diet quality and smoking status are reported in Table 1. In the total population, the mean ± SD of DQI-I and DQI-R were 63.41 ± 7.98 and 71.20 ± 12.61, respectively. Based on both DQI-I and DQI-R, individuals with higher vs. lower diet quality showed a higher proportion of men, and had higher mean age, education level, FBS, TG, SBP levels, and energy intake. Individuals with higher DQI-I had lower levels of HDL-C and employment rate. Also, those with higher DQI-R had higher BMI and higher physical activity levels compared with those with lower DQI-R. Compared to non-smokers, current smokers had significantly a higher percentage of men and TG levels, while mean age, BMI, physical activity, and HDL-C levels were significantly lower in the current smokers compared to non-smokers. Moreover, the energy intake was higher, and both quality diet scores were lower for current smokers than non-smokers.

**Table 1:** Baseline characteristics of the study participants for cardiovascular disease incidents and all-cause mortality based on diet quality and smoking status (n = 5048).

Characteristics	DQI-I			DQI-R			Smoking status		
	Lower	Higher	*P*-value	Lower	Higher	*P*-value	Non-smokers	Current smokers	*P*-value
Male (%)	39.9	49.8	0.001	42.5	47.1	0.001	38.8	86.2	0.001
Age (year)	45.1 ± 10.6	47.6 ± 11.2	0.001	45.5 ± 10.8	47.2 ± 11.1	0.001	46.5 ± 11.1	45.6 ± 9.8	0.040
BMI (kg/m^2^)	28.0 ± 4.6	28.1 ± 4.5	0.148	27.8 ± 4.6	28.3 ± 4.5	0.001	28.2 ± 4.5	27.1 ± 4.4	0.001
PA (MET/min/wk)	1208 (333–2656)	1190 (334–2560)	0.145	1125 (333–2500)	1250 (392–2737)	0.001	1205 (396–2525)	1000 (59–3334)	0.001
Education level (higher than diploma, %)	21.7	24.7	0.020	21.6	24.8	0.012	23.3	22.4	0.603
Employment status (employed, %)	88.3	85.9	0.004	88.1	86.4	0.079	87.5	85.4	0.141
FBS (mg/dl)	97 ± 26	100 ± 28	0.001	97 ± 26	99 ± 27	0.001	99 ± 27	98 ± 28	0.461
Serum TC (mg/dl)	194 ± 38	195 ± 37	0.428	194 ± 37	194 ± 38	0.932	194 ± 38	194 ± 37	0.719
Serum TG (mg/dl)	128 (90–181)	139 (97–199)	0.001	130 (90–184)	136 (96–195)	0.001	130 (91–187)	149 (105–213)	0.001
Serum HDL-C (mg/dl)	45.5 ± 11.1	44.8 ± 10.9	0.030	44.9 ± 10.9	45.3 ± 11.1	0.168	45.8 ± 11.0	40.2 ± 9.56	0.001
SBP (mmHg)	113 ± 16	117 ± 17	0.001	114 ± 17	116 ± 17	0.001	115 ± 17	114 ± 16	0.099
Energy intake (kcal)	2220 ± 736	2400 ± 681	0.001	2078 ± 716	2535 ± 637	0.001	2294 ± 710	2420 ± 736	0.001
FHCVD (%)	23.0	22.3	0.543	22.9	22.5	0.708	22.8	21.8	0.563
DQI-I	56.9 ± 5.2	69.8 ± 4.1	0.001	58.1 ± 6.6	68.5 ± 5.4	0.001	63.5 ± 7.9	62.1 ± 8.1	0.001
Variety	15.4 ± 3.2	16.8 ± 2.2	0.001	15.2 ± 3.3	17.0 ± 2.0	0.001	16.1 ± 2.8	16.2 ± 3.0	0.389
Adequacy	30.7 ± 4.02	33.5 ± 1.8	0.001	30.2 ± 3.8	34.0 ± 1.2	0.001	32.1 ± 3.3	31.9 ± 3.6	0.056
Moderation	9.78 ± 4.59	16.0 ± 4.0	0.001	11.2 ± 5.6	14.4 ± 4.5	0.001	13.0 ± 5.2	11.8 ± 5.8	0.001
Overall balance	1.00 ± 1.59	3.42 ± 2.13	0.001	1.44 ± 1.98	3.01 ± 2.22	0.001	2.23 ± 2.26	2.22 ± 2.24	0.860
DQI-R	62.9 ± 11.0	79.5 ± 7.5	0.001	60.8 ± 9.1	81.2 ± 5.2	0.001	71.5 ± 12.5	69.2 ± 12.4	0.001
Dietary diversity	5.84 ± 1.39	6.35 ± 1.10	0.001	5.56 ± 1.31	6.62 ± 1.00	0.001	6.09 ± 1.27	6.15 ± 1.35	0.265
Dietary moderation	6.03 ± 1.28	6.70 ± 0.99	0.001	6.09 ± 1.34	6.63 ± 0.96	0.001	6.40 ± 1.17	6.10 ± 1.29	0.001

*BMI* body mass index, *PA* physical activity, *FBS* fasting blood sugar, *TC* total cholesterol, *TG* triglycerides, *HDL-C* high-density lipoprotein cholesterol, *SBP* systolic blood pressure, *FHCVD* family history of premature CVD, *DQI-I* diet quality index-international, *DQI-R* diet quality index-revised.

Values are reported as mean ± standard deviation for normally distributed variables, median (interquartile range) for non-normally distributed variables, and percentage for categorical variables.

P-values were obtained by independent sample t-test for normally distributed continuous variables, the Mann–Whitney U test for non-normally distributed variables, and chi-square for categorical variables.

Supplementary Table S1 displays the baseline characteristics of the study participants recruited for the study of HTN. The mean age ± SD of the participant's age was 38.25 years, and 42.6% were men. The total population had the mean ± SD of DQI-I and DQI-R of 62.43 ± 8.03 and 70.00 ± 12.81, respectively. Considering the DQI-I and DQI-R, participants with higher diet quality, had a higher percentage of men, higher mean age and BMI, higher physical activity and education level, higher energy intake, and exhibited higher levels of FBS, TC, TG, and SBP. According to the DQI-I, participants with higher diet quality had a lower HDL-C level. Compared to non-smokers, current smokers were more likely to be male, had a higher age, higher physical activity level, higher levels of TC, TG, SBP, and higher energy intake, however, they had lower HDL-C, lower scores of DQI-I and its components including variety and moderation, and also lower DQI-R score and its moderation score.

After a mean follow-up of 9.0 years, 357 cases of CVDs (7.1%), 54 cases of stroke (1.1%), and 131 cases of mortality (2.6%) were ascertained. Also, considering the HTN outcome, 1032 cases of HTN (18%) were recorded after a mean follow-up period of 7.8 years. Our results indicated the robust accuracy of the multivariable models in predicting the incidence of the investigated outcomes. The Harrel’s C index scores for the associations between smoking and HTN (0.792), smoking and stroke (0.880), smoking and CVDs (0.823), smoking and mortality (0.847), DQI-I and HTN (0.791), DQI-I and stroke (0.895), DQI-I and CVDs (0.823), DQI-I and mortality (0.846), DQI-R and HTN (0.791), DQI-R and stroke (0.895), DQI-R and CVDs (0.823), and DQI-R and mortality (0.849) signify their predictive capability. Considering the interaction between diet quality and smoking, the multivariable models exhibited good predictive power concerning the incidence of HTN (DQI-I = 0.791, DQI-R = 0.791), stroke (DQI-I = 0.887, DQI-R = 0.887), CVDs (DQI-I = 0.823, DQI-R = 0.823), and all-cause mortality (DQI-I = 0.849, DQI-R = 0.851).

Table 2 represents the HR and 95% CI for cardiovascular events, all-cause mortality, and HTN across quartiles of DQIs. The higher DQI-I and DQI-R scores were associated with a higher risk of total cardiovascular events in the crude model but after accounting for covariates in model 1 and model 2, the associations became non-significant. The hazard of stroke decreased significantly across quartiles of DQI-I and DQI-R in both adjusted models. Compared with those in the first quartile of DQI-I and DQI-R, the fully adjusted HRs (95% CIs) were 0.37 (0.17, 0.82) and 0.33 (0.15, 0.72), respectively. The hazard of the all-cause mortality also decreased across the quartiles of DQI-I and DQI-R in the adjusted models; the fully adjusted HR (95% CI) in those in the highest quartile compared to first quartile was 0.57 (0.34, 0.97) for DQI-I and 0.51 (0.30, 0.86) for DQI-R. The hazard of HTN increased significantly across quartiles DQI-I (*P* trend = 0.001) and DQI-R (*P* trend = 0.003) in the unadjusted model but by inclusion of covariates in models 1 and 2 the associations became non-significant.

**Table 2:** Hazard ratio (95% confidence intervals) for cardiovascular events, all-cause mortality, and hypertension based on the quartiles of dietary quality indices.

Variables	Quartiles of dietary quality indices				
	Q1	Q2	Q3	Q4	*P* trend
*Cardiovascular events*					
DQI-I					
Follow-up time (year)	8.6	8.6	8.8	8.7	
Cases/populations	73/1262	80/1262	91/1262	113/1262	
Crude model	Ref	1.04 (0.75, 1.44)	1.24 (0.91, 1.69)	1.47 (1.09, 1.98)	0.004
Model 1	Ref	0.88 (0.63, 1.22)	1.14 (0.83, 1.56)	1.10 (0.81, 1.49)	0.262
Model 2	Ref	0.87 (0.62, 1.20)	1.12 (0.81, 1.53)	1.04 (0.77, 1.42)	0.423
DQI-R					
Follow-up time (year)	8.7	8.7	8.7	8.6	
Cases/populations	78/1262	80/1262	87/1262	112/1262	
Crude model	Ref	0.98 (0.72, 1.35)	1.09 (0.80, 1.49)	1.39 (1.03, 1.86)	0.015
Model 1	Ref	0.88 (0.64, 1.22)	0.92 (0.67, 1.26)	1.05 (0.78, 1.42)	0.531
Model 2	Ref	0.91 (0.66, 1.26)	0.91 (0.66, 1.25)	1.04 (0.76, 1.40)	0.680
*Stroke*					
DQI-I					
Follow-up time (year)	8.8	8.9	9.1	9.0	
Cases/populations	18/1262	14/1262	10/1262	12/1262	
Crude model	Ref	0.81 (0.40, 1.65)	0.58 (0.26, 1.27)	0.64 (0.30, 1.36)	0.199
Model 1	Ref	0.63 (0.30, 1.31)	0.49 (0.22, 1.09)	0.40 (0.18, 0.83)	0.024
Model 2	Ref	0.61 (0.29, 1.26)	0.47 (0.21, 1.03)	0.37 (0.17, 0.82)	0.015
DQI-R					
Follow-up time (year)	8.9	8.9	9.0	9.0	
Cases/populations	19/1262	13/1262	11/1262	11/1262	
Crude model	Ref	0.71 (0.34, 1.45)	0.55 (0.25, 1.19)	0.59 (0.28, 1.26)	0.146
Model 1	Ref	0.57 (0.27, 1.19)	0.40 (0.18, 0.88)	0.35 (0.16, 0.75)	0.006
Model 2	Ref	0.59 (0.28, 1.22)	0.38 (0.17, 0.83)	0.33 (0.15, 0.72)	0.004
*All-cause mortality*					
DQI-I					
Follow-up time (year)	8.8	9.0	9.1	9.1	
Cases/populations	35/1262	28/1262	36/1262	32/1262	
Crude model	Ref	0.83 (0.49, 1.40)	1.03 (0.63, 1.69)	0.89 (0.53, 1.48)	0.845
Model 1	Ref	0.66 (0.39, 1.12)	0.87 (0.53, 1.44)	0.59 (0.35, 0.99)	0.112
Model 2	Ref	0.66 (0.38, 1.12)	0.85 (0.52, 1.40)	0.57 (0.34, 0.97)	0.089
DQI-R					
Follow-up time (year)	9.0	9.0	9.0	9.0	
Cases/populations	37/1262	28/1262	36/1262	30/1262	
Crude model	Ref	0.72 (0.43, 1.22)	0.91 (0.56, 1.48)	0.77 (0.46, 1.28)	0.466
Model 1	Ref	0.62 (0.37, 1.06)	0.68 (0.41, 1.12)	0.50 (0.30, 0.84)	0.018
Model 2	Ref	0.67 (0.39, 1.13)	0.66 (0.40, 1.08)	0.51 (0.30, 0.86)	0.016
*Hypertension*					
DQI-I					
Follow-up time (year)	7.9	7.7	7.8	7.6	
Cases/populations	218/1430	266/1430	242/1430	306/1430	
Crude model	Ref	1.23 (1.03, 1.48)	1.11 (0.92, 1.33)	1.41 (1.18, 1.69)	0.001
Model 1	Ref	1.14 (0.95, 1.37)	1.01 (0.83, 1.21)	1.17 (0.97, 1.40)	0.191
Model 2	Ref	1.12 (0.94, 1.35)	0.99 (0.82, 1.19)	1.15 (0.96, 1.38)	0.244
DQI-R					
Follow-up time (year)	7.9	7.7	7.7	7.7	
Cases/populations	218/1430	254/1430	270/1430	290/1430	
Crude model	Ref	1.19 (0.99, 1.43)	1.26 (1.05, 1.51)	1.30 (1.09, 1.56)	0.003
Model 1	Ref	1.08 (0.90, 1.30)	1.10 (0.92, 1.33)	0.97 (0.81, 1.17)	0.689
Model 2	Ref	1.09 (0.90, 1.31)	1.09 (0.91, 1.31)	0.96 (0.80, 1.16)	0.590

*DQI-I* diet quality index-international, *DQI-R* diet quality index-revised.

Model 1, was adjusted for sex, age, systolic blood pressure, fasting blood sugar, serum cholesterol, serum triglyceride, high-density lipoprotein cholesterol, energy intake, BMI, and physical activity. Model 2 additionally adjusted for medication use, employment status, education level, and family history of premature CVD.

All diet quality indices were constructed according to each 1000 kcal of energy intake.

**Figure 1:**
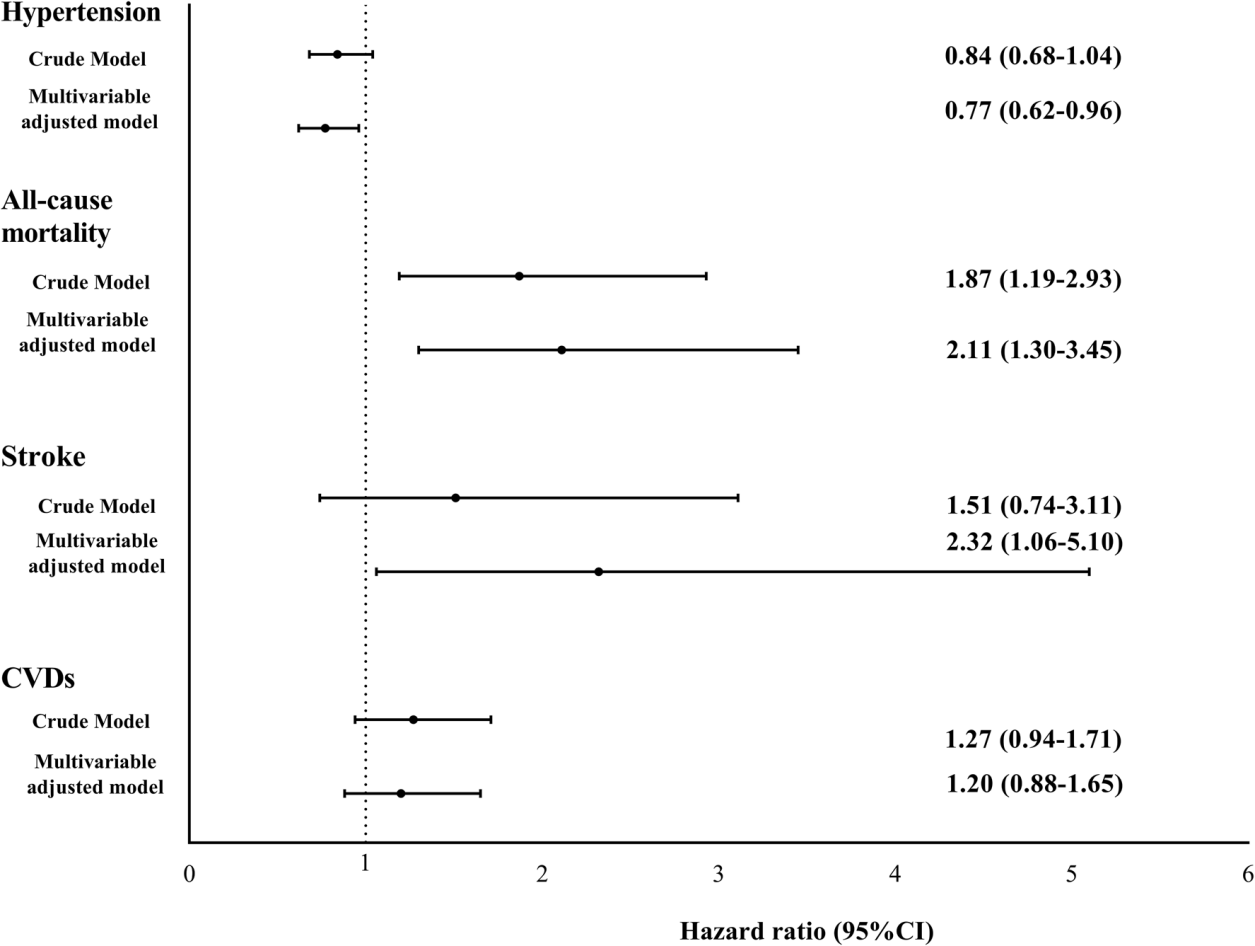
The hazard ratio and 95% confidence interval (CI) for the incidence of hypertension, all-cause mortality, stroke and cardiovascular diseases (CVDs) among current smokers vs. non-smokers (reference group): Tehran Lipid and Glucose Study.

Figure 1 indicates the hazard ratio for incidence of CVDs, stroke, mortality, and HTN in current smokers compared with non-smokers. In the crude model, the risk of all-cause mortality was significantly higher in the current smokers than non-smokers [HR 1.87, 95% CI (1.19–2.93)]; the association remained significant in the final adjusted model [HR 2.11; 95% CI (1.30–3.45)]. A significantly increased risk of stroke was also observed in the current smokers than the non-smokers after accounting for all potential covariates [HR 2.32; 95% CI (1.06–5.10)]. The risk of HTN was significantly lower in the current smokers compared to non-smokers in the fully adjusted model [HR 0.77; 95% CI (0.62–0.96)].

Table 3 displays the HR (95% CI) for cardiovascular events, all-cause mortality, and HTN based on the combined effects of diet quality and smoking status. No significant interaction (*P* interaction > 0.05) was noted among the studied groups concerning cardiovascular events, all-cause mortality, and HTN. Compared to current smokers with poor diet quality, the hazard (95% CI) of stroke was 0.23 (0.08, 0.63) in adjusted model 1 and 0.24 (0.08, 0.66) in the adjusted model 2 in non-smokers with good diet quality based on DQI-I. A lower risk of stroke was also observed in non-smokers with good diet quality based on DQI-R compared to current smokers with poor diet quality in the adjusted models. The risk of all-cause mortality was significantly lower in both non-smokers’ groups with higher or lower diet quality compared to the current smokers with poor diet quality in the crude model and after adjusting for potential covariates. The hazard of HTN was not significantly different across the combined groups.

**Table 3:** Hazard ratio (95% confidence intervals) for cardiovascular events, all-cause mortality, and hypertension based on the combined effects of diet quality and smoking status.

Variables	Hazard ratio (95% confidence interval)			
	Smoker-lower diet quality	Smoker-higher diet quality	Nonsmoker-lower diet quality	Nonsmoker-higher diet quality
*Cardiovascular events*				
DQI-I				
Cases/populations	28/370	26/268	125/2154	178/2256
Crude model	Ref	1.36 (0.78, 2.36)	0.77 (0.50, 1.17)	1.03 (0.68, 1.56)
Model 1	Ref	1.22 (0.70, 2.12)	0.82 (0.53, 1.27)	0.98 (0.64, 1.51)
Model 2	Ref	1.28 (0.74, 2.23)	0.86 (0.56, 1.33)	0.99 (0.64, 1.53)
DQI-R				
Cases/populations	29/394	25/244	129/2130	174/2280
Crude model	Ref	1.47 (0.84, 2.54)	0.82 (0.54, 1.24)	1.02 (0.68, 1.53)
Model 1	Ref	1.18 (0.68, 2.05)	0.87 (0.56, 1.33)	0.91 (0.59, 1.39)
Model 2	Ref	1.20 (0.69, 2.09)	0.90 (0.58, 1.38)	0.90 (0.59, 1.38)
*Stroke*				
DQI-I				
Cases/populations	6/370	3/268	26/2154	19/2256
Crude model	Ref	0.69 (0.17, 2.77)	0.68 (0.28, 1.67)	0.47 (0.18, 1.18)
Model 1	Ref	0.49 (0.12, 2.01)	0.40 (0.15, 1.04)	0.23 (0.08, 0.63)
Model 2	Ref	0.54 (0.13, 2.19)	0.45 (0.17, 1.16)	0.24 (0.08, 0.66)
DQI-R				
Cases/populations	6/394	3/244	26/2130	19/2280
Crude model	Ref	0.80 (0.20, 3.21)	0.73 (0.30, 1.79)	0.49 (0.19, 1.24)
Model 1	Ref	0.47 (0.11, 1.94)	0.42 (0.16, 1.09)	0.20 (0.07, 0.57)
Model 2	Ref	0.47 (0.11, 1.95)	0.45 (0.17, 1.17)	0.20 (0.07, 0.57)
*All-cause mortality*				
DQI-I				
Cases/populations	16/370	10/268	47/2154	58/2256
Crude model	Ref	0.83 (0.36, 1.89)	0.45 (0.25, 0.82)	0.53 (0.29, 0.94)
Model 1	Ref	0.65 (0.28, 1.49)	0.38 (0.20, 0.70)	0.37 (0.20, 0.68)
Model 2	Ref	0.76 (0.33, 1.75)	0.43 (0.23, 0.81)	0.40 (0.22, 0.75)
DQI-R				
Cases/populations	16/394	10/244	49/2130	56/2280
Crude model	Ref	0.95 (0.41, 2.18)	0.51 (0.28, 0.92)	0.53 (0.30, 0.95)
Model 1	Ref	0.61 (0.26, 1.43)	0.41 (0.22, 0.77)	0.32 (0.17, 0.61)
Model 2	Ref	0.66 (0.28, 1.53)	0.46 (0.24, 0.86)	0.34 (0.18, 0.63)
*Hypertension*				
DQI-I	58/373	45/289	426/2487	503/2571
Cases/populations	Ref	0.92 (0.61, 1.38)	1.06 (0.80, 1.40)	1.22 (0.92, 1.61)
Crude model	Ref	0.90 (0.60, 1.35)	1.20 (0.90, 1.60)	1.23 (0.92, 1.64)
Model 1	Ref	0.89 (0.59, 1.34)	1.21 (0.91, 1.62)	1.23 (0.92, 1.64)
Model 2				
DQI-R				
Cases/populations	61/390	42/272	411/2470	518/2588
Crude model	Ref	0.86 (0.57, 1.30)	1.00 (0.76, 1.32)	1.21 (0.92, 1.59)
Model 1	Ref	0.81 (0.54, 1.23)	1.17 (0.89, 1.54)	1.17 (0.89, 1.55)
Model 2	Ref	0.79 (0.52, 1.20)	1.17 (0.88, 1.55)	1.17 (0.88, 1.54)

*DQI-I* diet quality index-international, *DQI-R* diet quality index-revised.

Model 1, was adjusted for sex, age, systolic blood pressure, fasting blood sugar, serum cholesterol, serum triglyceride, high-density lipoprotein cholesterol, energy intake, BMI, and physical activity. Model 2 additionally adjusted for medication use, employment status, education level, and family history of premature CVD.

All diet quality indices were constructed according to each 1000 kcal of energy intake.

## Discussion

The current study revealed that a high-quality diet was linearly associated with a lower risk of stroke and mortality. Compared to non-smokers, current smokers had an higher risk of stroke, CVDs, and mortality, and a lower risk of HTN. Additionally, non-smokers with higher diet quality had a lower risk of stroke compared to current smokers with lower diet quality. Non-smokers experience a lower risk of mortality than current smokers with lower diet quality. Notably, the lower risk for stroke and all-cause mortality was more pronounced in non-smokers with higher diet quality than in those with lower diet quality.

The similarities in the associations between the DQI-I and the DQI-R with the studied outcomes are likely attributable to similarities in their food and nutrient components. In general, higher DQI-I and the DQI-R scores indicate a healthier diet, characterized by a variety of food choices, adequate intake of fruits, vegetables, grains, and micronutrients, and suitable percentages of total energy intake for total fat, saturated fats, and high-calorie foods. Additionally, the DQI-R considers moderation of alcohol consumption, which makes it valuable in populations where alcohol use is either excessive or contraindicated. Although both indices evaluate an approximately equal number of dietary components, the DQI-I encompasses a broader spectrum of dietary aspects compared to the DQI-R. In contrast to the DQI-R, the DQI-I assesses the adequacy of fiber, protein, and vitamin C intake, as well as the balance in ratios between macronutrients and fatty acids.

Despite the limited number of studies examining the link between diet quality, as assessed by the DQIs, and the incidence of CVDs and all-cause mortality, our findings contribute additional evidence supporting the role of overall diet quality in the prevention of CVDs outcomes. In a study conducted within the framework of the Korean National Health and Nutrition Examination Surveys, it was observed that, based on DQI-I, individuals with CVDs exhibited a lower diet quality in comparison to those without CVDs^24^. Abdurahman et al. reported that a lower DQI-I score was linked to a higher risk of metabolic syndrome and its components, particularly hypertriglyceridemia, and reduced HDL-C levels among obese adults^25^. A cross-sectional study conducted by Shiraseb et al. revealed an inverse association between the DQI-I score and serum inflammatory biomarkers^19^. However, a cross-sectional study by Daneshzad et al. revealed no significant association between DQI-I score and cardiovascular disease risk factors in type 2 diabetic women, possibly due to low statistical power. Of note, it has been suggested that different ratings of DQIs for evaluating diet quality in different populations, such as diabetic patients, be created^26^.

Current smokers seem to exhibit a lower risk of HTN compared to non-smokers. Previous studies suggest that the lower risk of HTN in current smokers is attributed to higher levels of physical activity and lower weight of smokers. Khodamradi et al., following a 20-year follow-up, implied that the lower risk of HTN among smokers may be linked to their increased participation in work-related activities^21^. Besides, smoking can impact the hypothalamus, leading to appetite suppression, reduced dietary intake, and elevated catecholamine levels. This results in higher energy consumption in peripheral tissues and, ultimately, a lower body weight for smokers^27^. However, smoking causes a temporary increase in blood pressure, which returns to normal levels after 20 min of smoking^10^. According to data from the CARDIA (Coronary Artery Risk Development in Young Adults) study, the detrimental effects of smoking on blood pressure changes and pulse pressure (as calculated by subtracting DBP from SBP) are more pronounced than those of blood pressure levels alone. Notably, cigarette smoking can cause stiffness in large elastic arteries, like the aorta. This stiffness disrupts the ability of vessels to adjust to pressure changes during systole and diastole. Although there was no significant difference between blood pressure levels in current and non-smokers, the acute increase in blood pressure coupled with the progression of atherosclerosis may amplify the likelihood of unfavorable outcomes for smokers^28^.

The risk of stroke in comparison to other CVDs events, was significantly higher among current smokers. Findings from the Atherosclerosis Risk in Communities (ARIC) study indicate relative risks for lacunar and cardioembolic strokes in current smokers of 2.30 and 1.94, respectively^29^. In addition, Kawachi et al. demonstrated that, within the framework of the Nurse's Health Study, nurses who smoke are almost three times more likely to experience a stroke^30^. The elevated risk associated with smoking and stroke is attributed to factors like increased atherosclerotic plaque formation, heightened thrombosis, reduced cerebral perfusion, facilitation of aneurysm formation and rupture, structural damage to the arterial wall, carboxyhemoglobinemia, heightened platelet aggregability, and elevated fibrinogen levels^31^. Additionally, in animal models, a constituent of environmental tobacco smoke like 1,3-butadiene, can accelerate atherosclerosis, further linking smoking to CVDs risk^10^.

Previous studies have presented contrasting perspectives regarding the interaction effects of diet and smoking on health outcomes. One perspective suggests that, due to smokers' weakened nutritional and clinical status, a healthy diet is expected to have more pronounced effects on smokers than non-smokers^32^. In addition, in alignment with our study's results, smoking emerged as an effect modifier in the association between diet quality and NCDs. In other words, smoking might diminish the potential beneficial impact of a high-quality diet. Geng et al. reported an increased risk of CVDs mortality in individuals who were genetically prone to smoking and who also had lower DASH (dietary approach to stop hypertension) scores^7^. Moreover, Margetts et al. suggested that the risk of oxidative damage is elevated in smokers due to a combination of insufficient nutrient intake and the impact of smoking on nutrient metabolism^33^. Of note, smoking and a low-quality diet contribute to CVDs development through shared pathways like endothelial dysfunction and inflammation^34^. While increased TG and decreased HDL-C levels are the main characteristics of metabolic syndrome in smokers^35^, previous studies have established a direct association between adherence to a high-quality diet, assessed by the DQI-I, and higher serum HDL-C levels^20^. A recent study by Noruzzadeh et al. suggests that among current smokers, individuals with higher diet quality experience a lower risk of all-cause mortality compared to current smokers with lower diet quality^36^. Additionally, the study found that this lower risk associated with higher diet quality was more pronounced among former smokers^36^. Although some studies emphasize the essential role of nutrition in overall health, they suggest that diet may not influence the relationship between smoking and disease. Li and colleagues suggested that the severity of asthma symptoms in smokers was not influenced by the quality of their diet^37^. In contrast, a high-quality diet, as measured by the Alternative Healthy Eating Index 2010, was linked to a decrease in the severity of asthma symptoms in nonsmokers^37^. Millen et al. suggested that smokers who adopt heart-healthy dietary modifications may have certain advantages, but their risk of CVDs and all-cause mortality remains higher compared to non-smokers who also follow a heart-healthy diet^6^. Of note, DQIs are a measure of overall dietary quality in the general population, but considering the specific health condition of smokers, different ratings of DQIs or other dietary indices designed to correct these conditions may be more suitable in preventing CVDs incidence and all-cause mortality. It is crucial to acknowledge that our results may be susceptible to residual confounding from smoking-related factors, including smoking intensity and duration.

This study has notable strengths, including an extended follow-up duration, an investigation of the combined impact of diet quality and smoking, the use of validated and reproducible FFQ to collect dietary data, and control for various potentially confounding variables. Nevertheless, this study has considerable limitations. Like all observational studies, it may be susceptible to measurement bias. Additionally, due to the lack of available data, alcohol consumption was not incorporated as a food component in the computation of the DQI-R. Last, as an observational study, it cannot fully eliminate the impact of all potential residual confounding variables.

Based on our findings, it appears that smoking may counteract the benefits of a high-quality diet within the general population. Furthermore, existing literature suggests that the dietary habits of current smokers could influence their mortality risk. Given the limitations of this study, future studies could explore the interaction between diet quality and smoking across diverse populations. Employing alternative interaction indices like the relative excess risk due to interaction (RERI) and synergy index (SI) may provide deeper insights into their combined impact on health outcomes. Furthermore, employing restricted cubic splines (RCS) analysis to assess the relationship between smoking intensity (measured by the number of cigarettes per day) and duration (pack-years) and health-related outcomes could yield more precise estimates of the impact of smoking on health risks. Given the observed interaction between higher diet quality and smoking regarding health outcomes, future nutrition studies should acknowledge smoking as a significant confounding variable. Also, considering the detrimental impact of smoking on overall mortality and the incidence of CVDs, strategies such as smoking cessation programs, smoking reduction initiatives, and the implementation of smoking bans in public areas can prove effective in alleviating the smoking-related burden of diseases.

In conclusion, following a high-quality diet can be associated with a lower risk of stroke and all-cause mortality, however, these beneficial effects may be eliminated by the adverse impact of smoking. Further research in different populations is warranted to examine the individual and combined effects of dietary quality indices and smoking on the risk of NCDs.

## Methods

### Study population

The current investigation was carried out within the Tehran Lipid and Glucose Study (TLGS) outline. The TLGS is an ongoing population-based research initiative designed to identify risk factors for NCDs and advocate for healthier lifestyles. Since 1999, six examinations, involving 15,005 participants who undergo routine physical examinations, laboratory tests, and updates to their medical histories every three years, have been conducted^38^.

In the third examination of the TLGS (2006–08), 3686 individuals were chosen at random for dietary assessment. In the subsequent fourth examination (2009–2011), 7956 individuals were randomly selected and participated in the dietary assessment. For the present study, participants with complete dietary intake data from the third examination and those newly enrolled in the fourth examination were considered. Notably, participants from the third examination who exhibited under-reporting or over-reporting of energy intake (≤ 800 kcal/day and ≥ 4200 kcal/day, respectively) were excluded (n = 233). However, if their energy intake fell within the normal range at the fourth examination (n = 100), their dietary data from the fourth examination were considered baseline dietary data. After excluding individuals with over- or under-reports of energy intake in the fourth examination (n = 502), a total of 8914 participants with complete dietary intake data were included.

**Figure 2:**
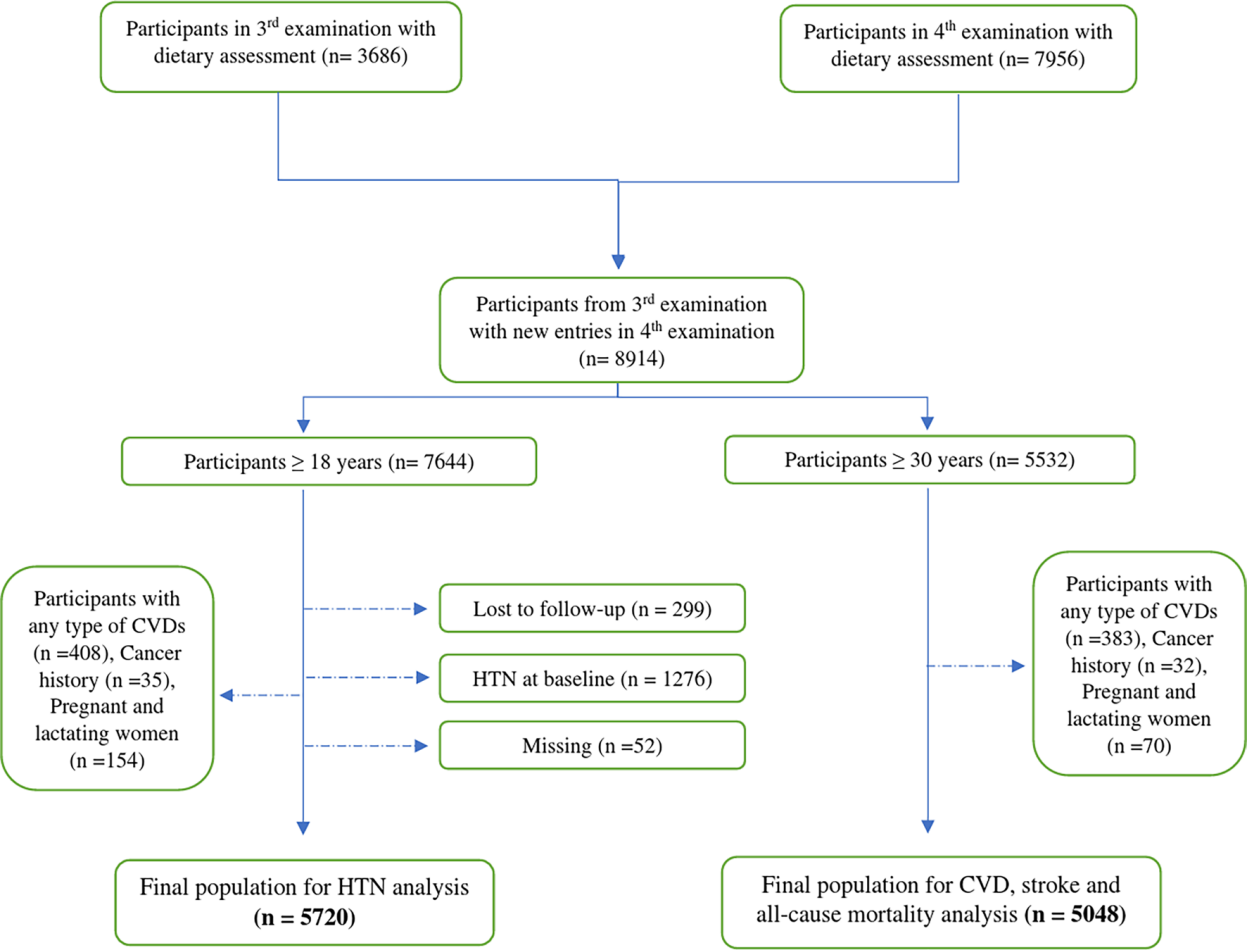
Diagram of the study participants and follow-up.

For the HTN outcome, exclusions were made for individuals aged < 18 years (n = 1270), individuals with any type of CVDs (n = 408), history of cancer (n = 35), pregnant or lactating women (n = 154), individuals with missing data on HTN status (n = 52), and those who were hypertensive at baseline (n = 1276). It is worth noting that some participants may belong to more than one exclusion category. Additionally, 299 participants were lost to follow-up, resulting in 5720 participants remaining for the final analysis of the HTN incident (Fig. 2).

Concerning the incidence of CVDs, and all-cause mortality, among the 5532 individuals aged ≥ 30 years, exclusions were made for those with any type of CVD (n = 383), a history of cancer (n = 32), pregnancy or lactation (n = 70) at the baseline of the study. Of note, some participants may belong to more than one exclusion category. Ultimately, a total of 5048 participants, free of CVDs were included in the study (Fig. 2) and were followed up until March 20, 2018, for the analysis of CVDs, and all-cause mortality.

### Dietary assessment

Nutrition specialists collected dietary intake data by employing a validated semi-quantitative food frequency questionnaire (FFQ) comprising 168 items^39^. This questionnaire assessed the frequency of participants' food consumption over the preceding year, categorizing responses into daily, weekly, or monthly frequencies. To ensure accuracy and consistency in the data analysis, portion sizes originally expressed in household measurements were converted into grams. The nutritional values of the food items were determined using the United States Department of Agriculture (USDA) food composition table (FCT)^40^. In instances where local foods were not listed in the USDA FCT, the Iranian FCT^41^ was utilized.

### Demographic and clinical measurements

To obtain demographic information, including age, sex, medication intake, and social and economic status, face-to-face interviews were conducted by trained interviewers using a standardized questionnaire. Annual phone follow-ups were carried out by the research team to monitor new medical incidents. In cases requiring further information, a physician conducted additional inquiries through home visits or reviews of medical records.

The participants' physical activity levels were assessed using the validated modifiable activity questionnaire (MAQ)^42^. This questionnaire, divided into leisure and work-related sections, assessed the duration and frequency of various activities undertaken in the past year. The total physical activity was computed in metabolic equivalent minutes per week (MET-min/week).

Blood pressure was measured using a calibrated mercury sphygmomanometer, following a 15-min rest period. A trained doctor recorded two blood pressure readings at 30-s intervals, and the average of these readings was considered the individual's blood pressure.

The height and weight of the participants were measured using a standardized procedure. To ensure accuracy, participants removed their shoes and wore light clothing during the measurements. A stadiometer was utilized for height measurements, while a calibrated weighing scale was used to record weight. Body mass index (BMI) was calculated by dividing individuals’ weight in kilograms by the square of their height in meters.

Laboratory analyses were conducted utilizing kits provided by Pars Azmon Inc. Participants' venous blood samples were obtained during a fasting period of 12–14 h, between 7:00 and 9:00 a.m. The measurement of serum HDL-C involved the isolation of apoB-containing lipoproteins through phosphotungstic acid precipitation. Serum triglyceride (TG) and total cholesterol (TC) levels were determined using an enzymatic colorimetric method, employing cholesterol esterase and cholesterol oxidase for TG, and glycerol phosphate oxidase for TC. Fasting blood sugar (FBS) was measured using the glucose oxidase technique and an enzymatic colorimetric method. Samples were analyzed only when they met internal quality control standards. The coefficients of variation were 2.2% for serum glucose, 2% (intra-assay) and 0.5% (inter-assay), for TC, and 1.6% (intra-assay), and 0.6% (inter-assay) for TG.

Also, a premature family history of CVDs was defined as the occurrence of any CVDs incidents in first-grade female family members aged < 65 years, or first-grade male family members aged < 55 years^43^.

### Exposure definition

#### Current and non-smokers

Participants who smoke daily or occasionally, or who have quit smoking for less than a year, are considered current smokers^44^. Non-smokers are defined as individuals who have quit smoking for more than a year or have no smoking history.

#### Dietary Quality Index-International (DQI-I)

To calculate the DQI-I, we utilized the approach outlined by Kim et al.^45^. This method evaluates four primary components: 1) Variety in the intake of food groups, encompassing meat, poultry, fish, eggs, dairy, vegetables, fruits, grains, and legumes, as well as diversity within protein sources (scored on a scale of 0–20 points); 2) Adequacy in the consumption of 8 dietary components including vegetables, fruits, grains, fiber, protein, and micronutrients including iron, calcium, and vitamin C (scored on a scale of 0–40 points); 3) Moderation in the consumption of total fat, saturated fats, cholesterol, sodium, and free-calorie foods (scored on a scale of 0–30 points); and 4) Overall balance in the ratio of macronutrients (carbohydrate: protein: fat) and fatty acids (polyunsaturated fats: monounsaturated fats: saturated fats), (scored on a scale of 0–10 points). Scores can range from 0 to 100, with higher scores indicative of a higher level of diet quality.

#### Dietary Quality Index-Revised (DQI-R)

The DQI-R comprises ten elements, four of which align with the original DQI (total fat, saturated fat, cholesterol, and calcium). Additionally, the DQI-R assesses grain, fruit, vegetable, iron, dietary moderation, and dietary diversity. Each element is assigned a rating on a scale from 0 to 10, contributing to a maximum cumulative score of 100. The moderation component involves managing simple sugars, discretionary fat, sodium, and dietary alcohol, while diversity encompasses a variety of grains, fruits, vegetables, meats, and dairy products in the diet^46^.

### Outcome definition

The studied outcomes involve CVDs, HTN, and all-cause mortality. CVDs were described as a combined measure involving incidents of coronary heart disease, stroke, or death due to cardiovascular causes. Events related to coronary heart disease included confirmed cases of myocardial infarction (determined by diagnostic electrocardiogram, and biomarkers), possible myocardial infarction (identified by positive electrocardiogram findings, symptoms or signs of a heart attack, and absence of biomarkers; or positive electrocardiogram findings and uncertain biomarkers), and coronary heart disease confirmed through angiography. Stroke was characterized by the presence of newly developed neurological impairment lasting at least 24 h.

According to the Joint National Committee on Prevention, Detection, Evaluation, and Treatment of High Blood Pressure (JNC 7) guidelines, HTN was diagnosed if an individual’s systolic blood pressure (SBP) was ≥ 140 mmHg, diastolic blood pressure (DBP) was ≥ 90 mmHg, or if they were on medication for HTN management^47^.

In the case of all-cause mortality, an authorized local physician collected information from hospital records or death certificates. A committee, comprising key professionals including a chief researcher, an internist, an endocrinologist, a cardiologist, an epidemiologist, and the individual responsible for compiling outcome data, evaluated the results obtained.

### Statistical analyses

The statistical analysis was performed utilizing IBM SPSS Statistics (Version 26.0, IBM Corp., Armonk, NY, USA). The characteristics of the study population are reported as percentages for categorical variables, means ± standard deviation (SDs) for normally distributed variables, and medians (interquartile ranges) for non-normally distributed variables. Comparisons between the groups, including those with higher and lower diet quality or current and non-smokers, were performed using the chi-square test for categorical variables, independent sample t-test for normally distributed continuous variables, and the Mann‒Whitney U test for non-normally distributed variables. Our study did not encounter missing data.

Cox proportional hazard regression was employed to explore the associations of DQI quartiles and smoking status (non-smokers vs current smokers) with the incidence of HTN, stroke, CVDs, and all-cause mortality. The duration of follow-up was calculated from the enrollment date in the study until the occurrence of the first CVDs or mortality event or until the last follow-up date. Additionally, for the incidence of HTN, the follow-up duration was defined as the midpoint between the date of the follow-up visit where HTN was initially identified and the last follow-up visit preceding the diagnosis.

Additionally, an interaction analysis was conducted based on the joint classification of DQIs (median; higher/lower) and smoking status (current smokers/non-smokers). For the interaction analysis, four groups were created: 1) current smokers with lower diet quality, 2) current smokers with higher diet quality, 3) non-smokers with lower diet quality, and 4) non-smokers with higher diet quality. The hazard ratios (HRs) and their associated 95% confidence intervals (CIs) for each outcome were determined across the categories considering “current smokers with poor diet quality” as a reference group. Variables that had significant associations with CVDs and HTN according to the univariate analysis (*P* < 0.05), were included in the statistical analyses as confounding factors. Model 1 was adjusted for sex, age, SBP, FBS, serum TC, serum TG, serum HDL-C, energy intake, BMI, and physical activity. Additionally, Model 2 was adjusted for medication use, employment status, education level, and family history of premature CVDs. The fitness of the multivariable models was determined using Harrel’s C index^48^. To obtain *P* values for interactions, smoking status, diet quality status, and their interaction terms were included in the unadjusted Cox model. The results include HR (95% CI) for each comparison. The proportional hazards assumption was checked using the graph of the log(−log(survival)) versus the log of survival time, and the assumption was satisfied (lines in the plots were parallel). All *P* values were derived from two-sided tests, and values less than 0.05 were considered to indicate statistical significance.

### Ethics approval and consent to participate

The study followed the Declaration of Helsinki by obtaining informed consent from participants and receiving approval from the Ethics Committee of the Iran University of Medical Sciences (IR.IUMS.REC.1402.138).

### Supplementary Information

Supplementary Information.

## Data Availability

The datasets used and/or analyzed during the current study are available from the corresponding author upon reasonable request.
