# Supplementary material for: The interaction between diet quality and cigarette smoking on the incidence of hypertension, stroke, cardiovascular diseases, and all-cause mortality
Source: Sci Rep. 2024 May 29;14:12371. doi: 10.1038/s41598-024-62616-9 (PMC11137059; doi:10.1038/s41598-024-62616-9)
Supplement: Supplementary file 1 — Supplementary Information. [file 41598_2024_62616_MOESM1_ESM.docx]

| **Supplementary Table S1.** Baseline characteristics of the study participants for hypertension incidents based on diet quality and smoking status (n=5720) ^a,b^ | | | | | | | | | |
| --- | --- | --- | --- | --- | --- | --- | --- | --- | --- |
| **Characteristics** | **DQI-I** | | | **DQI-R** | | | **Smoking status** | | |
|  | **Poor** | **Good** | ***P*-value** | **Poor** | **Good** | ***P*-value** | **Non-smokers** | **Smokers** | ***P*-value** |
| Male (%) | 37.9 | 52.7 | 0.001 | 41.1 | 55.6 | 0.009 | 36.8 | 86.9 | 0.001 |
| Age (year) | 36.6 ± 12.5 | 39.9 ± 13.0 | 0.001 | 37.0 ± 12.7 | 39.6 ± 12.8 | 0.001 | 38.0 ± 13.0 | 40.1 ± 11.8 | 0.001 |
| BMI (kg/m^2^) | 26.3 ± 4.7 | 26.9 ± 4.5 | 0.001 | 26.2 ± 4.7 | 27 ± 4.5 | 0.001 | 26.6 ± 4.7 | 26.4 ± 4.5 | 0.254 |
| PA (MET/min/wk) | 1,071  (297-2,500) | 1,125  (333-2,541) | 0.004 | 989  (267-2,500) | 1,190  (333-2,619) | 0.001 | 1,073  (333-2,500) | 1,114  (119-3,426) | 0.001 |
| Education level (higher than diploma, %) | 25.3 | 29.9 | 0.001 | 25.5 | 29.5 | 0.001 | 27.7 | 24.8 | 0.110 |
| Employment status (employed, %) | 82.7 | 83 | 0.788 | 82.9 | 82.8 | 0.954 | 82.6 | 85 | 0.121 |
| FBS (mg/dl) | 92 ± 19 | 95 ± 22 | 0.001 | 92 ± 20 | 94 ± 21 | 0.001 | 93 ± 21 | 94 ± 22 | 0.407 |
| Serum TC (mg/dl) | 181 ± 37 | 185 ± 38 | 0.001 | 182 ± 38 | 184 ± 37 | 0.027 | 183 ± 37 | 186 ± 38 | 0.037 |
| Serum TG (mg/dl) | 106  (76-154) | 118  (82-171) | 0.001 | 107  (77-156) | 117  (82-170) | 0.001 | 109  (78-159) | 136  (91-191) | 0.001 |
| Serum HDL (mg/dl) | 46.3 ± 11.1 | 45.4 ± 11.2 | 0.004 | 45.8 ± 11.0 | 46.0 ± 11.3 | 0.386 | 46.5 ± 11.2 | 40.9 ± 9.62 | 0.001 |
| SBP (mmHg) | 107 ± 12 | 109 ± 12 | 0.001 | 107 ± 12 | 109 ± 12 | 0.001 | 108 ± 12 | 109 ± 13 | 0.022 |
| DBP (mmHg) | 72 ± 9 | 72 ± 9 | 0.787 | 72 ± 9 | 72 ± 9 | 0.371 | 72 ± 9 | 72 ± 9 | 0.722 |
| Energy intake (kcal) | 2,276 ± 748 | 2,425 ± 682 | 0.001 | 2,156 ± 733 | 2,574 ± 633 | 0.001 | 2,336 ± 717 | 2,456 ± 731 | 0.001 |
| FHCVD (%) | 19.0 | 19.2 | 0.883 | 18.9 | 19.3 | 0.700 | 19.0 | 20.1 | 0.486 |
| DQI-I | 55.9 ± 5.2 | 68.8 ± 4.2 | 0.001 | 57.6 ± 6.6 | 68.0 ± 5.4 | 0.001 | 62.5 ± 8.0 | 61.8 ± 7.9 | 0.047 |
| *Variety* | 15.4 ± 3.4 | 16.9 ± 2.3 | 0.001 | 15.3 ± 3.4 | 17.1 ± 2.0 | 0.001 | 16.1 ± 3.0 | 16.4 ± 3.0 | 0.040 |
| *Adequacy* | 30.5 ± 4.1 | 33.3 ± 2.9 | 0.001 | 30.1 ± 3.9 | 34.0 ± 1.2 | 0.001 | 31.9 ± 3.5 | 31.7 ± 3.6 | 0.180 |
| *Moderation* | 9.1 ± 4.6 | 15.3 ± 4.1 | 0.001 | 10.6 ± 5.6 | 14.0 ± 4.5 | 0.001 | 12.2 ± 5.3 | 11.5 ± 5.7 | 0.001 |
| *Overall balance* | 0.90 ± 1.54 | 3.33 ± 2.15 | 0.001 | 1.38 ± 1.96 | 2.96 ± 2.23 | 0.001 | 2.10 ± 2.24 | 2.16 ± 2.20 | 0.516 |
| DQI-R | 61.4 ± 11.02 | 78.4 ± 7.8 | 0.001 | 60.4 ± 9.3 | 81 ± 5.1 | 0.001 | 70.1 ± 12.8 | 68.9 ± 12.2 | 0.023 |
| *Dietary diversity* | 5.87 ± 1.42 | 6.39 ± 1.09 | 0.001 | 5.64 ± 1.33 | 6.70 ± 0.97 | 0.001 | 6.12 ± 1.29 | 6.21 ± 1.34 | 0.095 |
| *Dietary moderation* | 5.90 ± 1.29 | 6.62 ± 1.01 | 0.001 | 5.98 ± 1.34 | 6.57 ± 0.96 | 0.001 | 6.27 ± 1.21 | 6.11 ± 1.25 | 0.001 |
| Hypertension incidence (%) | 17.0 | 19.1 | 0.032 | 16.8 | 19.5 | 0.006 | 18.4 | 15.6 | 0.077 |
| **Abbreviations: BMI**, Body mass index**; PA**, Physical activity**; FBS**, Fasting blood sugar**; TC**, Total cholesterol**; TG**, Triglycerides**;** **HDL**, High-density lipoprotein cholesterol**; SBP**, Systolic blood pressure**; FHCVD**, Family history of premature CVD**;** **DQI-I**, Diet quality index-international**;** **DQI-R**, Diet quality index-revised.  ^a^ values are reported as mean ± standard deviation for normally distributed variables, median (interquartile range) for non-normally distributed variables, and percentage for categorical variables. ^b^ P-values were obtained by independent sample t-test for normally distributed continuous variables, the Mann-Whitney U test for non-normally distributed variables, and chi-square for categorical variables. | | | | | | | | | |
